# Personality and cortical architecture in premenstrual dysphoric disorder

**DOI:** 10.1007/s00737-026-01677-3

**Published:** 2026-03-09

**Authors:** Elise Bücklein-Ehlers, Manon Dubol, Birgit Derntl, Andreas Fallgatter, Inger Sundström-Poromaa, Erika Comasco

**Affiliations:** 1 Uppsala University, Uppsala, Sweden; 2 University of Tübingen, Tübingen, Germany; 3 German Center for Mental Health, Tuebingen, Germany; 4 Science for Life Laboratory, Uppsala, Sweden

**Keywords:** Brain, Cortex, Personality, PMDD, Symptom severity

## Abstract

**Purpose:**

Premenstrual dysphoric disorder (PMDD) has been associated with altered grey matter architecture in a trait-like manner. Personality traits, shaped largely by genetics and linked to depressive disorders, may relate to structural properties of the brain and could represent a potential factor mediating vulnerability for PMDD. However, these possible associations remain largely unexplored.

**Methods:**

The present study assessed personality traits in participants with PMDD and healthy controls, as well as how personality is related to symptom severity and cortical surface measures in PMDD. Healthy controls and patients completed the Swedish Universities Scale of Personality. Following prospective validation of the PMDD diagnosis, patients had their brain scanned with magnetic resonance imaging (MRI) during the symptomatic luteal phase of the menstrual cycle. Personality of controls and patients was compared using Mann-Whitney U tests. Associations of personality with symptom severity and brain surface parameters were tested through correlation analysis.

**Results:**

PMDD was associated with higher scores in neuroticism and aggressiveness. Aggressiveness was positively correlated with the severity of irritability/anger, though not significant after correcting for multiple testing. Regarding cortical structural measures, aggressiveness was negatively correlated with cortical complexity of the parahippocampal gyrus.

**Conclusion:**

Considering neuroticism and aggressiveness during screening for PMDD could contribute to the identification of risk factors and personalized treatment of females suffering from PMDD.

**Supplementary Information:**

The online version contains supplementary material available at 10.1007/s00737-026-01677-3.

## Article Highlights

- PMDD was associated with higher scores in neuroticism and aggressiveness.
- Aggressiveness positively correlated with irritability/anger.
- Aggressiveness negatively associated with parahippocampal gyrus complexity.
- Considering neuroticism and aggressiveness when assessing PMDD.

**Supplementary Information:**

The online version contains supplementary material available at 10.1007/s00737-026-01677-3.

## Introduction

Premenstrual Dysphoric Disorder (PMDD) is a mental condition associated with hormonal changes occurring during the menstrual cycle (Epperson et al. 2012). Core symptoms include depressed mood, anxiety, affective lability, and irritability or anger (Wittchen et al. 2002; Endicott et al. 2006; Yonkers et al. 2008; Epperson et al. 2012). PMDD is not associated with altered levels of ovarian hormones, but instead with fluctuations of these hormones across the menstrual cycle (Schmidt et al. 2017; Comasco et al. 2021; Rubinow and Schmidt 2025). Symptoms appear during the luteal phase of the menstrual cycle, peak during the late luteal phase, and usually subside a few days after the onset of menses (Epperson et al. 2012; Boron and Boulpaep 2017). The burden of PMDD extends across the reproductive lifespan and is associated with many comorbidities, including ADHD (Broughton et al. 2025), mood disorders (Bengi et al. 2025), and increased risk for peripartum depression (Schleimann-Jensen et al. 2025) as well as suicidal ideation (Wikman et al. 2022).

Considering the cyclicity of PMDD symptoms and that variation in brain structure has been observed during the menstrual cycle in healthy controls (Zsido et al. 2023), interest has been steered on understanding the neural underpinnings of PMDD. So far, differential activation as well as differential anatomy of limbic brain structures related to emotional and cognitive processing have been indicated as neural correlates of PMDD (Dubol et al. 2020). Regarding morphological features, in a large sample, Dubol et al. (2022a) found thinner cortico-limbic cortices in patients with PMDD when compared to healthy controls. Remarkably, these differences seem to represent traits rather than states, as confirmed by their presence during both the asymptomatic and symptomatic phases in patients with PMDD (Dubol et al. 2024), thus pointing to constitutive substrates of vulnerability.

A possible trait-like factor contributing to PMDD symptomatology and its neurobiology are personality traits, which are characteristic ways of thinking, feeling, and behaving. High scores of neuroticism are associated with major depression (Kendler 1993; Mulder 2002; Kotov et al. 2010) and postpartum depression (Puyané et al. 2022). Therefore, it is plausible that certain personality traits may increase the risk of developing PMDD. To date, only two studies have investigated personality traits in relation to premenstrual symptomatology yielding inconsistent results. Passive-aggressive personality traits have been associated with Late Luteal Phase Dysphoric Disorder (LLPDD, former PMDD) (Parry et al. 1996). Also, higher neuroticism scores have been noted in individuals with PMDD, particularly in patients with the most severe symptoms (Gingnell et al. 2010). Since these studies were based on small samples, studies investigating associations between PMDD and personality traits in larger PMDD samples are needed.

Largely determined by genetics (Bouchard and McGue 2003), personality may be associated with determined anatomical brain features, which could represent a neural vulnerability for PMDD symptomatology. Structural properties of the brain have indeed been associated with personality, e.g., trait anger (Sorella et al. 2022), neuroticism (Liu et al. 2021), and anxiety (Saviola et al. 2020). 

So far, the biological underpinnings of the relationship between personality traits and PMDD have virtually not been investigated, with only one study investigating genetic functional variations (Gingnell et al. 2010). The relationship between personality and grey matter architecture in PMDD remains unstudied. Likewise, there are to date no studies on the relationship between personality, PMDD symptoms and grey matter properties such as thickness and gyrification.

The present study therefore sought to investigate symptom characteristics from a neurobiologically informed perspective and investigate how personality traits of patients with PMDD are related to symptom severity, as well as to cortical surface measures, collected during the symptomatic luteal phase. The following questions were addressed: (i) Do personality traits differ between patients with PMDD and controls? (ii) How do personality traits relate to PMDD symptom severity? And (iii) How does personality relate to cortical surface measures in PMDD patients?

## Materials and methods

The study was approved by the ethical review board of Uppsala (Dnr. 2016/184 and 2016/312 and 2013/161). Participants were recruited through local newspapers and social media and were compensated for their participation. They received written and oral information about the study and had the possibility to ask questions before giving their written consent. The study has been described in Dubol et al. (2022) and Lundin et al. (2017).

### Participants and procedure

#### Participants with PMDD

In total, 75 females with PMDD were recruited. As described in Dubol et al. (2022), participants with PMDD were between 22 and 46 years old and had regular menstrual cycles (between 25 and 35 days). Exclusion criteria for participation were ongoing mental health problems as assessed by the Mini International Neuropsychiatric Interview (MINI) (Sheehan et al. 1998), present substance abuse, previous diagnosis for mental disorders including attempted suicide, or treatment with psychotropic medication during the previous 3 months. Of note, participants with a past history of depressive or anxiety disorders were admitted into the study if they were in remission for at least two years. Females who had severe medical conditions, had been treated with hormonal contraceptives or steroids during the previous 3 months, were breastfeeding, pregnant, or planned a pregnancy were also excluded.

The participants first attended a screening visit to gather demographic data and evaluate eligibility. To confirm PMDD diagnosis according to the criteria of the Diagnostic and Statistical Manual of Mental Disorders (DSM-5), (American Psychiatric Association 2013) participants recorded PMDD symptoms for two consecutive cycles using a smartphone application with the Daily Record of Severity of Problems (DRSP) (Endicott et al. 2006). After two months of scoring, participants were scheduled for a second meeting in the luteal phase of the menstrual cycle, during which MRI scans were performed.

#### Healthy controls (HC)

The sample of healthy participants was a combination of participants from two studies, *n* = 31 from the study described in the present article, and *n* = 147 from Lundin et al. (2017), resulting in a total of *n* = 178. Baseline procedures were similar for all participants.

### Psychometrics

The DRSP questionnaire describes the 11 psychological and physical DSM-5 symptoms of PMDD in 21 separate items and rated on a scale from 1 “not at all” to 6 “extreme”. To assess PMDD symptom severity, the DRSP sum score was calculated as the mean total DRSP score during the last five days of the premenstrual period (Endicott et al. 2006). Likewise, the scores for the four core symptoms (depression, anxiety, irritability and affective lability) were calculated as the mean score of the respective DRSP items during the last five days of the premenstrual period.

### Personality

The Swedish universities Scales of Personality (SSP) from Gustavsson et al. (2000) was administered to participants at the screening visit. It is a self-rating questionnaire, based on the Karolinska Personality Scales (KSP) (Schalling et al. 1987). Participants rate 91 items on a scale from 1 to 4, where 1 is “does not apply at all” and 4 is “applies completely”. The items form 13 scales, which are grouped into three major factors: a neuroticism factor (containing the scales Somatic Trait Anxiety, Psychic Trait Anxiety, Stress Susceptibility, Lack of Assertiveness, Embitterment, and Mistrust), an aggressiveness factor (containing the scales Trait Irritability, Verbal Trait Aggression, Physical Trait Aggression, and the inversed value of Social Desirability), and an extraversion factor (containing the scales Impulsiveness, Adventure Seeking, and the inversed value of Detachment) (Gustavsson et al. 2000). The 13 scale scores were calculated, transformed into standardized t-scores, and adjusted for sex according to Gustavsson et al. (2000) . The three major factors were calculated according to Võhma et al. (2010).

### MRI acquisition

All participants with PMDD were scanned in the luteal phase with a 3.0 Tesla whole-body scanner (Achieva dStream, Philips Medical Systems, Best, The Netherlands) equipped with a 32-channel head coil as described in Dubol et al. (2022). Anatomical 3D-T1-weighted whole-brain scans were carried out using an MPRAGE sequence with the following parameters: repetition time (TR) = 8.3 ms, echo time (TE) = 3.8 ms, matrix size 256 × 256, flip angle = 8°, 220 slices, acquisition time: 3:50 min. Resulting images had a 0.94 × 0.94 × 1 mm voxel size with a dimension of 256 × 256 × 220.

### MRI data preprocessing

MRI preprocessing was carried out using the Statistical Parametric Mapping software (SPM12, Wellcome Trust Centre for Neuroimaging) implemented in MATLAB R2022b (MathWorks, Natick, MA, USA), as described in Dubol et al. (2022). Briefly, images were manually reoriented, then segmented using the CAT12 preprocessing pipeline (http://dbm.neuro.uni-jena.de/cat). This includes a projection-based thickness estimation, allowing the computation of cortical thickness and cortical surface (Dahnke et al. 2013), along with partial volume correction and correction for sulcal blurring and sulcal asymmetries. A gyrification index was extracted based on absolute mean curvature (Luders et al. 2006). Cortical complexity, reflecting cortical folding patters that capture the complexity of the cortical surface geometry, was estimated using fractal dimension analysis. Sulcal depth and cortical thickness measures were also extracted. Surface meshes were re-parametrized into a common coordinate system using spherical maps for inter-subject comparisons (Yotter et al. 2011). Finally, all cortical surface measures were resampled and smoothed, using a FWHM Gaussian kernel of 15 mm for cortical thickness, and 20 mm for all other parameters. After the preprocessing procedure, the CAT12 quality control module for surface data was applied to exclude outliers. All further analyses were based on the extracted surface measures.

### Regions of interest

For the extracted surface measures, we defined regions of interest (ROIs) that were associated with group differences or correlations with symptom severity in previous studies on PMDD (Dubol et al. 2020, 2022a, 2022). The following ROIs were defined using the Desikan-Killiany (DK-40) atlas: the caudal ACC, rostral ACC, insula, PHG, LgG, FuG, and eight prefrontal regions (superior frontal, pars opercularis, pars orbitalis, pars triangularis, rostral middle frontal, caudal middle frontal, lateral orbitofrontal and medial orbitofrontal).

### Statistical analyses

Initially, 75 participants with PMDD were recruited. One person was excluded due to a brain tumor (*n* = 1). Due to missing personality questionnaires (*n* = 8), the analysis of the first question comparing personality traits in participants with PMDD and HC included *n* = 66 participants. Due to missing personality questionnaires (*n* = 8), and incomplete DRSP ratings during the menstrual cycle when the MR scan was performed (*n* = 10), the second question regarding how personality traits relate to PMDD symptom severity included *n* = 56 participants. Due to invalid MRI data (*n* = 3), missing personality questionnaires (*n* = 8), and females reporting no menses (*n* = 3) for question three on how personality relates to cortical surface measures in PMDD patients, *n* = 60 participants were included.

#### Comparison of personality between PMDD participants and healthy controls

To investigate differences in personality between PMDD participants and healthy controls, SSP scores for the personality factors neuroticism, aggressiveness and extraversion were compared. Because most scales showed signs of not being normally distributed, the non-parametric Mann-Whitney U test was used. Of those factors that differed significantly between groups, subscales were also compared in a second step. Results were corrected for multiple testing using the Bonferroni correction.

#### Correlations between personality scores and PMDD symptom severity

To investigate how personality is related to PMDD symptom severity, DRSP scores (total score, depression, anxiety, emotional lability and irritability scores) were correlated with the three personality factors (neuroticism, aggressiveness and extraversion) and subscales using Pearson’s partial correlation, controlling for age. The personality factors and subscales that were significantly correlated with DRSP scores were then used for the following analyses.

#### Correlations between personality and grey matter structure within ROIs

The personality factors that were significantly correlated with DRSP scores were, in turn, correlated with brain surface parameters in the PMDD group, such as cortical thickness, gyrification index, cortical complexity and sulcal depth of ROIs using Pearson’s partial correlation. In all correlations between personality and structural ROI data, total intracranial volume (TIV), age and body mass index (BMI) were included as covariates due to their potential influences on surface measures (Barnes et al. 2010; Zheng et al. 2019; García-García et al. 2019). All ROI-based analyses were carried out for each hemisphere separately. Statistical analyses were performed in R (R Core Team 2023) using the packages *Hmisc* (Harrell 2023) and *psych* (Revelle 2023).

## Results

**Fig. 1:**
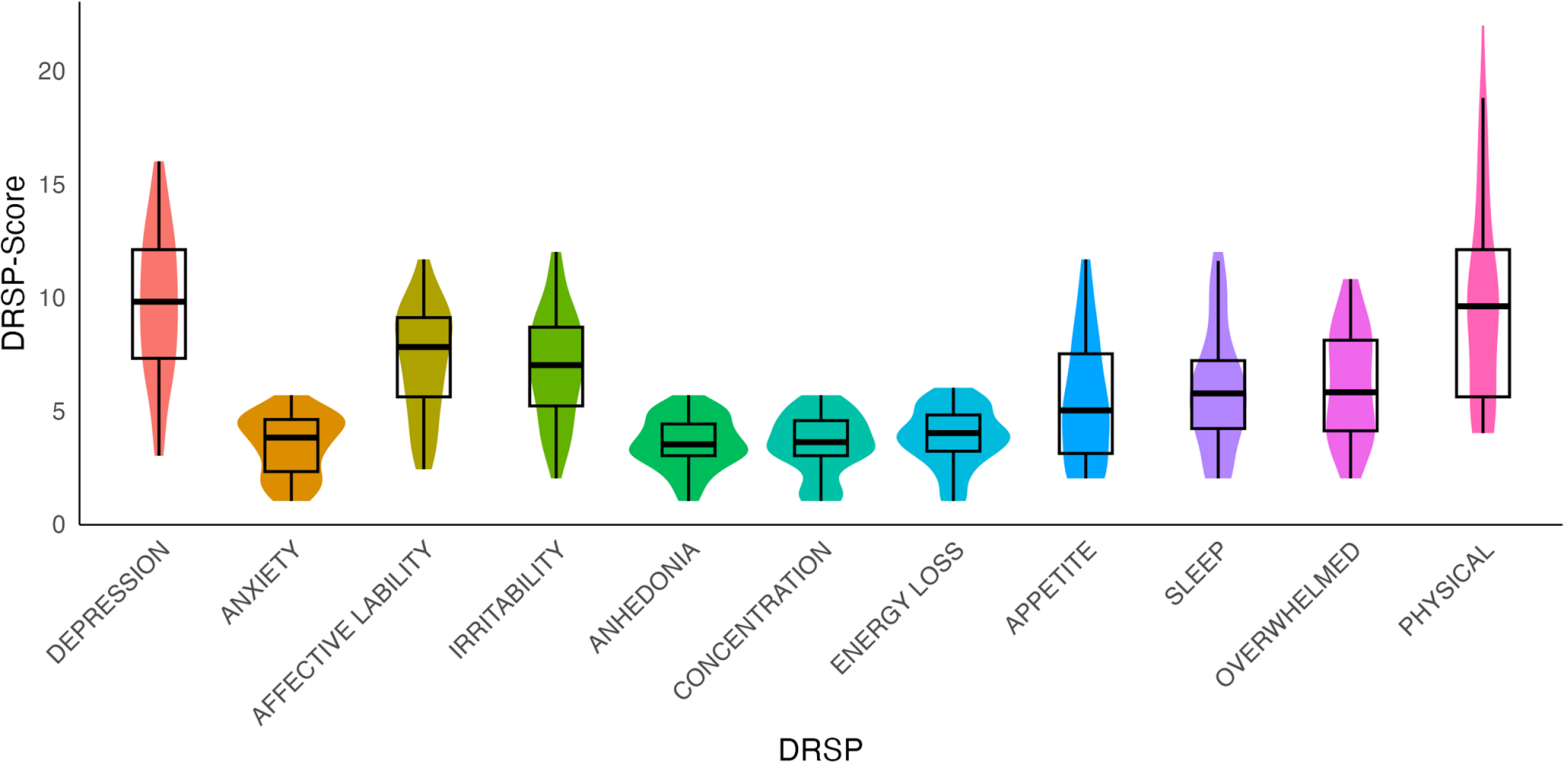
DRSP scores for PMDD. Violin plots and box plots for DRSP core and secondary symptoms for participants with PMDD.*Note.* DEPRESSION markedly depressed mood, ANXIETY markedly anxiety and tension, AFFECTIVE LABILITY marked affective lability, IRRITABILITY markedly irritability or anger, ANHEDONIA decreased interest in usual activities, CONCENTRATION difficulty concentrating, ENERGY LOSS lethargy and marked loss of energy, APPETITE change in appetite, SLEEP hypersomnia or insomnia, OVERWHELMED feeling overwhelmed or out of control, PHYSICAL physical symptoms (breast tenderness, breast swelling, muscle or joint pain)

Sample characteristics of participants with PMDD and healthy participants included in the analysis are presented in Table 1. The two groups differed in age, with the PMDD group being older than the healthy controls, as well as BMI, education level and parity. DRSP scores for all 11 DSM-5 symptoms are presented in Fig. 1.

**Table 1:** Participants characteristics

Characteristics	Mean (SD) or *n* (%)	
	PMDD *n* = 66	HC *n* = 178
Age (years)	34.7 (6.0)	25.2 (5.3)*
BMI	24.1 (4.1)	22.4 (2.7)*
Education level		*
High school	11 (16.9)	34 (18.6)
University	54 (83.0)	144 (81.4)
Nulliparous	28 (41.5)	138 (78.4)*
Nicotine use	13 (20.0)	19 (10.7)
Mental history		
Depressive disorder	26 (40.6)	37 (21.1)*
Anxiety disorder	10 (15.6)	30 (17.2)
**PMDD characteristics**		
Age at onset (years)	23.9 (6.5)	
Illness duration (years)^a^	10.8 (6.5)	
Previous PMDD treatment	52 (80.0)	
SSRI	42 (65.6)	
Hormonal	25 (38.5)	
Homeopathic	8 (12.9)	
Psychological	5 (8.1)	

^a^ Illness duration is based on self-reports. * difference between groups significant, *p* < 0.05. BMI body mass index, PMDD premenstrual dysphoric disorder, SD standard deviation, SSRI selective serotonin reuptake inhibitor

### Personality scores in patients with PMDD vs. healthy controls

**Fig. 2:**
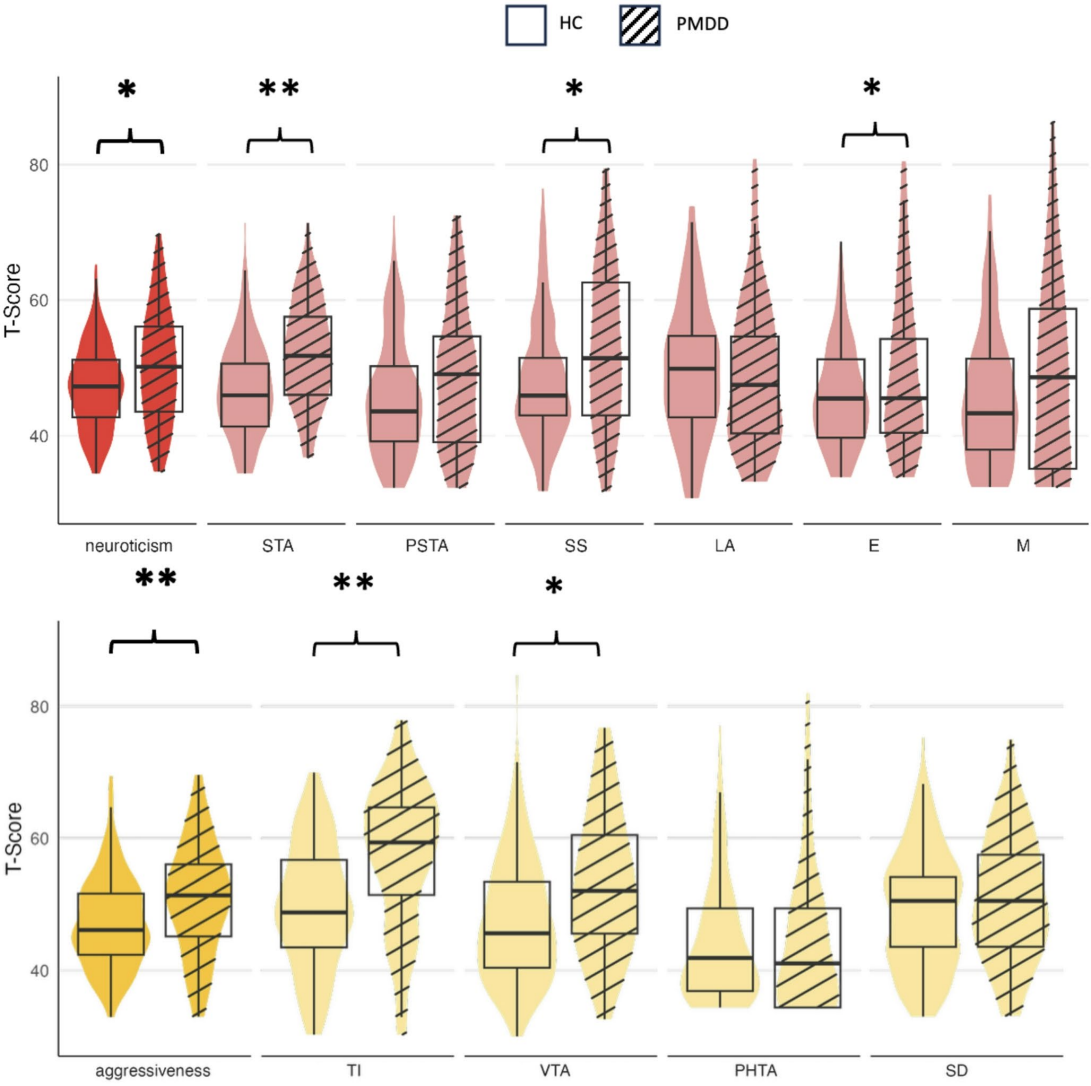
Neuroticism and aggressiveness scores for PMDD vs. HC. Violin and box plots for mean scores of the SSP factors neuroticism and aggressiveness and their subscales for healthy participants (HC) and participants with PMDD; *significant group difference, **significant after adjusting for multiple testing, *p*< 0.0042. *Note*. SSP Swedish university scales of personality, STA somatic trait anxiety, PSTA psychic trait anxiety, SS stress susceptibility, LA lack of assertiveness, E embitterment, M mistrust, TI trait irritability, VTA verbal trait aggression, PHTA physical trait aggression, SD reversed value for social desirability

PMDD was associated with significantly higher values for the neuroticism factor (PMDD: *median =* 50.2, HC: *median =* 47.1, *U* = 6269, *p* = 0.014), and the aggressiveness factor (PMDD: *median =* 51.4, HC: *median* = 47.3, *U* = 6576, *p* = 0.002). SSP factor scores on neuroticism, and aggressiveness as well as subscales are reported in Fig. 2. The neuroticism factor subscales Somatic Trait Anxiety (*U* = 7452.5, *p* < 0.001), Stress Susceptibility (*U* = 6247.5, *p* = 0.016), and Embitterment (*U* = 6210, *p* = 0.02), as well as the aggressiveness factor subscales Trait Irritability (*U* = 7372, *p* < 0.001) and Verbal Trait Aggression (*U* = 6441, *p* = 0.005) also differed significantly between groups. After correcting for multiple testing, Somatic Trait Anxiety and Trait Irritability remained significant. For more details, see Table A1 of the Appendix. These results remained virtually unchanged when assessing possible confounding effects of variables differing between groups (age, BMI, education and parity).

### Correlations between personality scores and PMDD symptom severity

**Fig. 3:**
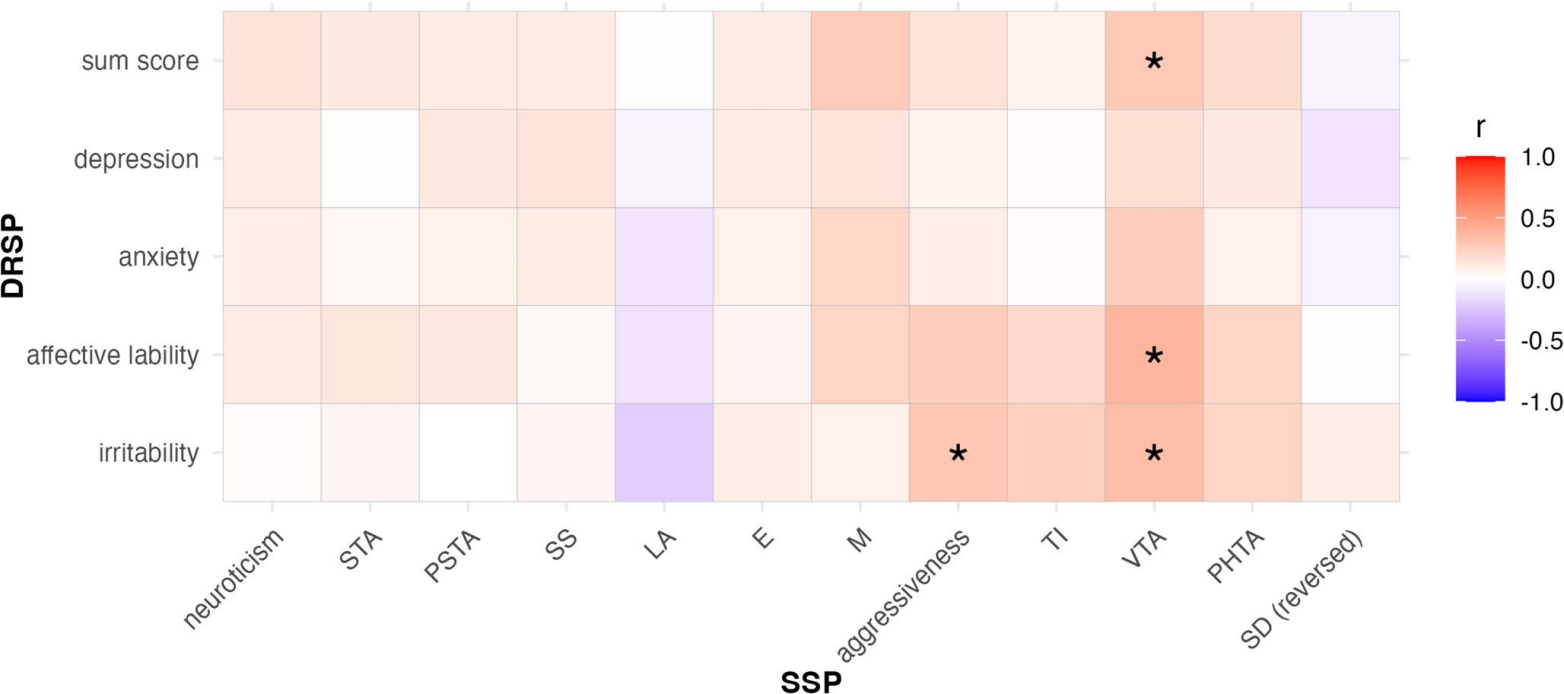
Correlations of symptom severity scores with personality scores for PMDD. Correlations of DRSP scores with personality factors neuroticism and aggressiveness and respective subscales for participants with PMDD. *p< 0.05, presented uncorrected for multiple testing. *Note*. DRSP daily record of severity of problems, SSP Swedish university scales of personality, STA somatic trait anxiety, PSTA psychic trait anxiety, SS stress susceptibility, LA lack of assertiveness, SD (reversed) reversed value for social desirability, E embitterment, TI trait irritability, M mistrust, VTA verbal trait aggression, PHTA physical trait aggression

Correlations between the personality factors neuroticism and aggressiveness (and their subscales) and the DRSP scores are presented in Fig. 3. DRSP total score was positively correlated with aggressiveness subscale Verbal Trait Aggression (*r* = 0.28, *p* = 0.05). DRSP irritability/anger scores were positively correlated with aggressiveness factor scores (*r* = 0.29, *p* = 0.04) and scores of the aggressiveness subscale Verbal Trait Aggression (*r* = 0.34, *p* = 0.02). DRSP affective lability scores were also positively correlated with scores of the aggressiveness subscale Verbal Trait Aggression (*r* = 0.38, *p* = 0.01). After correcting for multiple testing, none of the correlations remained significant.

### Correlations between personality scores and brain structure

**Fig. 4:**
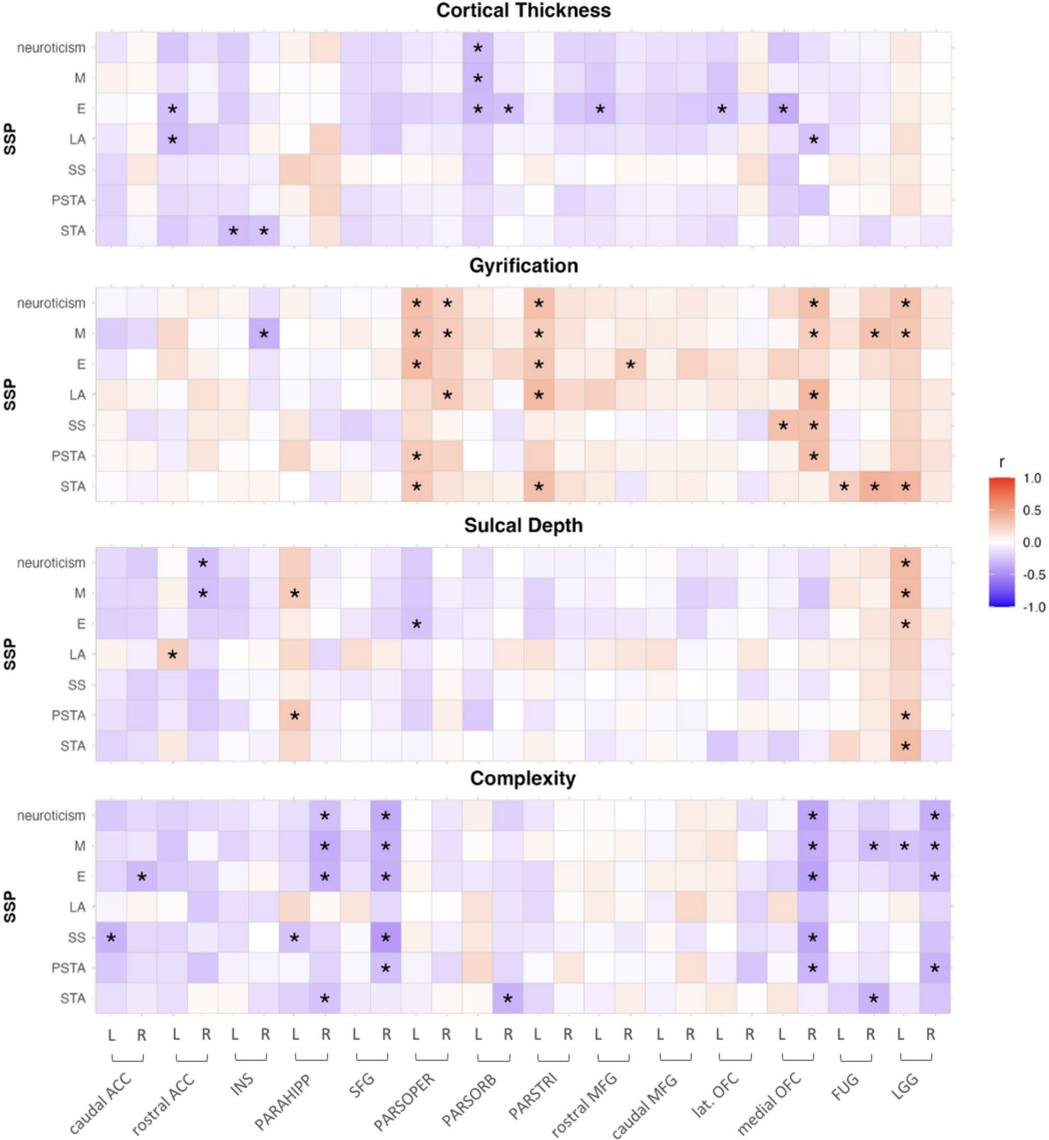
Correlations of neuroticism scores with surface measures in PMDD group. Correlations of neuroticism and 6 subscales with surface measures for thickness, gyrification, sulcal depth and complexity for participants with PMDD,* *p*< 0.05 after correcting for age, BMI and TIV.*Note.* SSP Swedish University Scales of Personality, M mistrust, E embitterment, LA lack of assertiveness, SS stress susceptibility, PSTA psychic trait anxiety, STA social trait anxiety, L left, R right, ACC anterior cingulate cortex, INS insula, PARAHIPP parahippocampal gyrus, SFG superior frontal gyrus, PARSOPER pars opercularis, PARSORB pars orbitalis, PARSTRI pars triangularis, MFG middle fusiform gyrus, lat. OFC lateral orbitofrontal cortex, FUG fusiform gyrus, LGG lingual gyrus

Correlations between the neuroticism factor scores and surface measures of cortical thickness, gyrification, sulcal depth and cortical complexity within ROIs are presented in Fig. 4. The strongest correlations were found between the complexity of the right superior frontal gyrus (*r* = −0.37, *p* = 0.004) and the right medial OFC (*r* = −0.39, *p* = 0.002), which were negatively correlated with the total score of neuroticism, as well as the complexity of the right SFG, which was also negatively correlated with the subscale Stress Susceptibility (*r* = −0.44, *p* = 0.00052). None of the correlations survived corrections for multiple testing (*p*_*adj*_ = 0.00029).

**Fig. 5:**
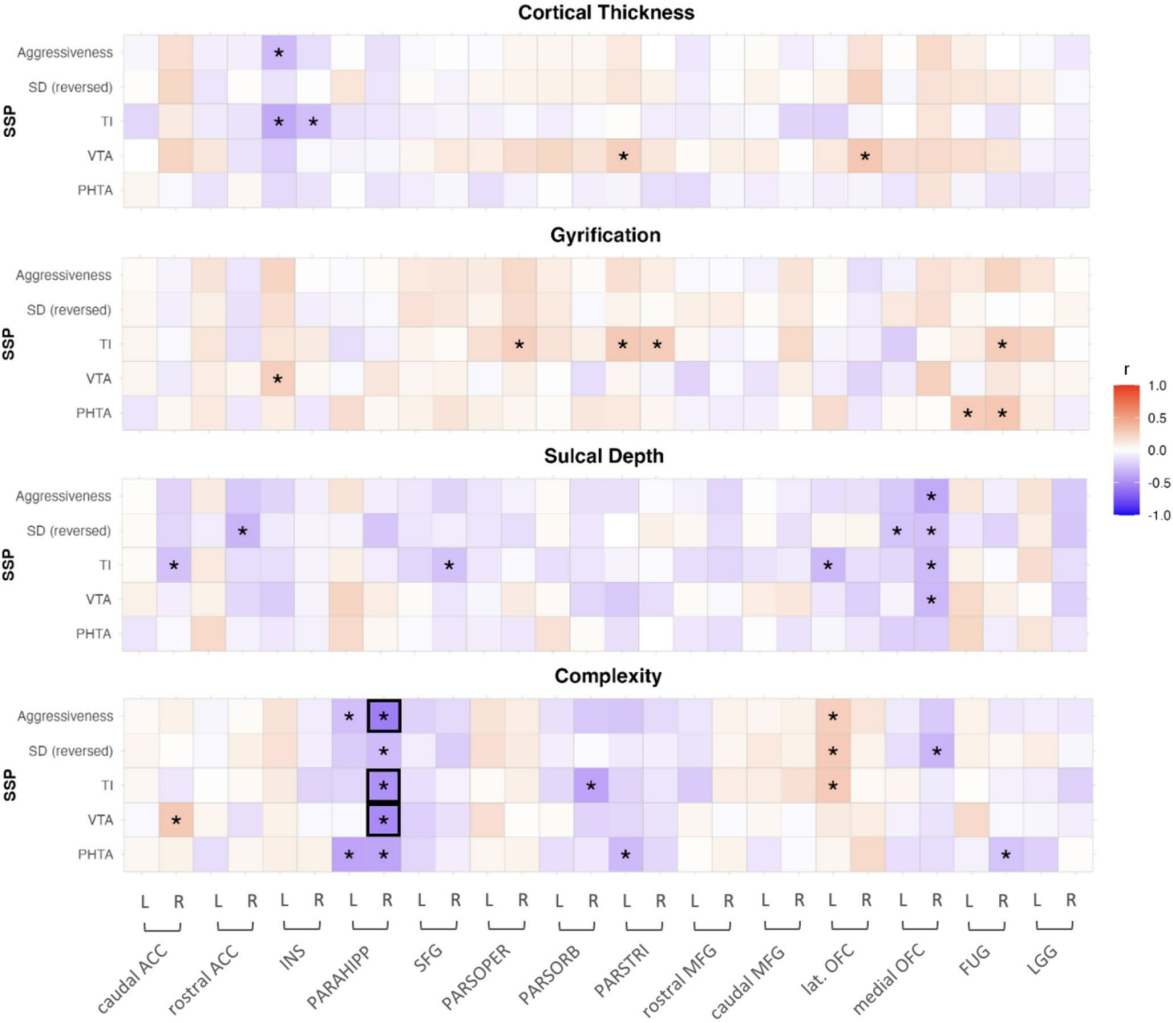
Correlations of aggressiveness scores with surface measures. Correlations of aggressiveness and 4 subscales with surface measures for thickness, gyrification, sulcal depth and complexity for participants with PMDD; * *p*< 0.05 after correcting for age, BMI and TIV, *p*< 0.00036, Bonferroni corrected.*Note.* SSP Swedish University Scales of Personality, SD social desirability, TI trait irritability, VTA verbal trait aggression, PHTA physical trait aggression, L left, R right, ACC anterior cingulate cortex, INS insula, PARAHIPP parahippocampal gyrus, SFG superior frontal gyrus, PARSOPER pars opercularis, PARSORB pars orbitalis, PARSTRI pars triangularis, MFG middle fusiform gyrus, lat. OFC lateral orbitofrontal cortex, FUG fusiform gyrus, LGG lingual gyrus

Correlations between the aggressiveness factor scores and surface measures of ROIs are presented in Fig. 5. Regarding aggressiveness, the strongest correlation was found between the complexity of the right parahippocampal gyrus and aggressiveness factor (*r* = −0.56, *p* = 0.00001) as well as subscales Trait Irritability (*r* = −0.48, *p* = 0.00018), and Verbal Trait Aggression (*r* = −0.51, *p* = 0.00005), which were all negatively associated. These correlations remained significant after correcting for multiple testing (*p*_*adj*_ = 0.00036).

## Discussion

The present findings demonstrate that females with PMDD have higher scores of neuroticism and aggressiveness in comparison with healthy controls. Additionally, the aggressiveness factor and the aggressiveness subscale Verbal Trait Aggression were positively correlated with the severity of irritability/anger in PMDD, even though the correlations did not survive correction. Furthermore, neuroticism and aggressiveness were associated with ROI-based surface measures. Particularly aggressiveness was negatively correlated with the complexity of the right parahippocampal gyrus.

The finding of higher neuroticism scores in PMDD aligns with previous research (Gingnell et al. 2010). Additionally, trait aggressiveness scores were higher in females with PMDD compared to healthy controls, which may not be surprising given that irritability/anger, one of the key symptoms of PMDD, can be a precursor to aggressive behavior. In line with results regarding higher trait aggressiveness in PMDD, we also found a positive correlation between the aggressiveness factor, as well as the subscale Verbal Trait Aggression, and PMDD symptom severity, particularly irritability/anger and affective lability. Even though these correlations did not survive correction for multiple testing, whether there is a causal relationship between PMDD symptoms and trait aggressiveness warrants further investigation. Interestingly, irritability/anger has been the symptom category that improved the most upon administration of a newly proposed treatment for PMDD with a selective progesterone receptor modulator (Comasco et al. 2021). This treatment, while reducing irritability, has also been associated with enhanced fronto-cortical activation during aggressive response to provocation in females with PMDD compared with placebo (Kaltsouni et al. 2021), suggesting greater top-down cognitive control over emotions. Indeed, participants receiving the placebo showed a negative correlation between aggressive behavior and activation of the fronto-cortical cluster, further highlighting a plausible link between irritability/anger, aggression and these brain regions (Kaltsouni et al. 2021).

The study highlights aggressiveness as a central personality trait in PMDD as it is associated with luteal phase irritability/anger and grey matter morphology differences, notably in the parahippocampal gyrus, which is involved in emotional processing, particularly through connections with the amygdala and the hippocampus in the limbic system. Interestingly, previous research regarding correlations between symptom severity and cortical metrics in a largely overlapping sample has found that the complexity of the parahippocampal gyrus was negatively associated with the severity of luteal phase irritability and affective lability (Dubol et al. 2022), supporting its relevance in understanding the neural basis of PMDD and its connection to personality. Our finding therefore supports previous research showing that the most consistent brain alterations in PMDD involve cortical structures related to emotional and cognitive processing (Dubol et al. 2020).

This study benefits from a well-characterized and relatively large sample of females assessed during the mid to late luteal phase with confirmed PMDD diagnosis based on daily prospective symptom ratings, while cycle phase confirmation was achieved through hormone assessments and cycle mapping. Considering the trait-like nature of the investigated variables, the present study focuses on the luteal phase of the menstrual cycle. Indeed, personality traits are not expected to vary between phases (Gnambs 2014), and recent within-subject studies suggest that anatomical MRI alterations in PMDD are present across cycle phases (Dubol et al. 2024; Stiernman et al. 2025). To control for potential biases, BMI, age, and TIV were included as covariates in the analysis on neuroimaging metrics. Being an exploratory study, the aim was to test multiple associations to generate hypotheses for future research, albeit leading to a large number of tests. We also considered whether personality traits might mediate the associations between symptom severity and the cortical features found in the present study to be significantly associated with personality as a potential mechanism behind PMDD. However, because there was no significant association between the symptom severity of irritability and cortical features of those ROIs significantly associated with personality in this study, the assumptions for mediation were not fulfilled and therefore no mediation analysis was performed.

The relationship between personality traits, PMDD symptom severity, and brain structure suggests the relevance of personality for shaping both the experience and neurobiology of PMDD. Incorporating brief personality assessments – such as screening for aggressiveness or neuroticism – could help clinicians identify individuals who may benefit from more tailored interventions. For example, patients with heightened aggressiveness might respond well to targeted emotion-regulation strategies, while those with high neuroticism might benefit from cognitive-behavioral approaches. Awareness of personality-linked structural alterations may support more personalized pharmacological treatments, particularly regarding irritability. Overall, our findings support a biopsychosocial framework for PMDD that encourages individualized treatment rather than a purely hormone-focused one-size-fits-all approach.

## Supplementary Information

Below is the link to the electronic supplementary material.

Supplementary Material 1 (DOCX 22.3 KB)

## Data Availability

Data can be made available upon request to the corresponding author.
