# Supplementary material for: Personality and cortical architecture in premenstrual dysphoric disorder
Source: Arch Womens Ment Health. 2026 Mar 9;29(2):48. doi: 10.1007/s00737-026-01677-3 (PMC12971799; doi:10.1007/s00737-026-01677-3)
Supplement: Supplementary file 1 — Supplementary Material 1 (DOCX 22.3 KB) [file 737_2026_1677_MOESM1_ESM.docx]

**Appendix**

**Table A1**

*SSP neuroticism and aggressiveness and subscales comparison PMDD vs. HC*

|  | **PMDD** | | **HC** | |  |  |
| --- | --- | --- | --- | --- | --- | --- |
| **SSP factor / subscale** | **median** | **IQR** | **median** | **IQR** | **p** | **U** |
| Neuroticism | 50.2 | 12.5 | 47.1 | 8.5 | **0.014*** | 6269 |
| STA | 51.8 | 11.5 | 47.1 | 9.3 | **< 0.001**** | 7452.5 |
| PSTA | 49.1 | 15.6 | 45.6 | 11.1 | 0.175 | 5775 |
| SS | 51.4 | 19.6 | 47.9 | 10.4 | 0.016* | 6247.5 |
| LA | 47.5 | 14.3 | 49.5 | 12 | 0.302 | 4696 |
| E | 45.5 | 13.8 | 45.7 | 11.6 | 0.02* | 6210 |
| M | 48.6 | 23.6 | 45.5 | 13.4 | 0.743 | 5310.5 |
| Aggressiveness | 51.4 | 10.9 | 47.3 | 9.3 | **0.002**** | 6576 |
| TI | 59.4 | 15.2 | 49.3 | 13.2 | **< 0.001**** | 7372 |
| VTA | 52.1 | 14.9 | 47.4 | 13 | 0.005* | 6441 |
| PHTA | 41.1 | 15 | 43.9 | 12.5 | 0.611 | 5392 |
| SD (inverted) | 48.8 | 13.9 | 48.4 | 14 | 0.729 | 5318.5 |

*Note*. Using non-parametric Wilcoxon-test. * significant difference between groups p < 0.05; ** significant difference after adjusting for multiple testing, *p* < 0.0042; SSP Swedish university scales of personality, PMDD premenstrual dysphoric disorder, HC healthy control, IQR interquartile range (quartile 3 – quartile 1), STA somatic trait anxiety, PSTA psychic trait anxiety, SS stress susceptibility, LA lack of assertiveness, E embitterment, M mistrust, TI trait irritability, VTA verbal trait aggression, PHTA physical trait aggression, SD reversed value for social desirability.
